# Flower-Visiting Butterflies Avoid Predatory Stimuli and Larger Resident Butterflies: Testing in a Butterfly Pavilion

**DOI:** 10.1371/journal.pone.0166365

**Published:** 2016-11-15

**Authors:** Yuya Fukano, Yosuke Tanaka, Sayed Ibrahim Farkhary, Takuma Kurachi

**Affiliations:** 1 Graduate School of Agricultural and Life Sciences, The University of Tokyo, Tokyo, Japan; 2 Ueno Zoological Gardens, Tokyo Zoological Park Society, Tokyo, Japan; 3 Department of Veterinary Medicine, Faculty of Agriculture, Tokyo University of Agriculture and Technology, Tokyo, Japan; University of Western Australia, AUSTRALIA

## Abstract

The flower-visiting behaviors of pollinator species are affected not only by flower traits but also by cues of predators and resident pollinators. There is extensive research into the effects of predator cues and resident pollinators on the flower-visiting behaviors of bee pollinators. However, there is relatively little research into their effects on butterfly pollinators pr**o**bably because of the difficulty in observing a large number of butterfly pollination events. We conducted a dual choice experiment using artificial flowers under semi-natural conditions in the butterfly pavilion at Tama Zoological Park to examine the effects of the presence of a dead mantis and resident butterflies have on the flower-visiting behavior of several butterfly species. From 173 hours of recorded video, we observed 3235 visitations by 16 butterfly species. Statistical analysis showed that (1) butterflies avoided visiting flowers occupied by a dead mantis, (2) butterflies avoided resident butterflies that were larger than the visitor, and (3) butterflies showed greater avoidance of a predator when the predator was present together with the resident butterfly than when the predator was located on the opposite flower of the resident. Finally, we discuss the similarities and differences in behavioral responses of butterfly pollinators and bees.

## Introduction

The relationship between flowering plants and insect pollinators is one of the most important mutualisms in terrestrial ecosystems [1–4]. The flower-visiting behaviors of pollinators have been shaped by mutualistic interactions with flowering plants, and the flower-visiting behaviors and preference of pollinators are influenced by flower traits such as color, shape, and odor [4–7]. However, mutualism with flowers is not the only biotic factor affecting the behavior of pollinators. Other biotic interactions such as antagonistic and competitive interactions are also important for pollinators in the community network [8,9]. Thus, several types of cues from antagonists (e.g. predators) and competitors can also affect the flower-visiting behaviors of pollinators.

First, flower-visiting insects may face predation risks from ambush predators on and near the flowers [10–13]. Because predation events have a considerable impact on the fitness of pollinators, pollinators have evolved the ability to avoid several predator-related cues. Pollinators change their flower-visiting behavior in response to the presence of live, dead and model predators [11,14–17]. These predator avoidance behaviors might be achieved by avoiding any foreign objects on the flower [18]. Moreover, pollinators can avoid flowers containing cues of past predation events [15,19]. Abbot (2006) found that flowers containing a freshly killed bumblebee or scent of the killed bumblebee received fewer bumblebee visitations than control flowers. The avoidance of predation risks by pollinators can reduce plant reproductive success and result in disruption of the mutualistic interaction between flowering plants and pollinators [16,20,21].

Second, if most of the flower nectar and pollen is consumed by pollinators, avoiding flowers occupied by live resident pollinators might be adaptive behavior for flower-visiting pollinators. On the other hand, if the nectar and pollen are not limited resources, following and preference for resident pollinators can be advantageous by reducing the energy cost of flower-searching behavior [22]. While many studies have found that flower-visiting animals do not show spatial aggregation, suggesting avoidance of resident pollinators [23–25], others studies have reported that flower-visiting animals are attracted to resident pollinators when searching for novel flowers [26–28]. Kawaguchi et al. (2007) found that pollinators adjust their response to the presence of conspecific pollinators depending on their familiarity with the flowers they are visiting [29]. Their study showed that bumblebees avoided visiting familiar flowers when conspecific bumblebees were present, but that they preferred visiting unfamiliar flowers when conspecific bumblebees were present. Furthermore, the presence of conspecific pollinators can be used as an indicator of safety for pollinators that experienced predation. Dawson & Chittka (2014) demonstrated that bumblebees prefer to feed with conspecific bumblebees when they are presented with a previously predator-infested flower. That study suggests that bumblebees can integrate information about predators and resident conspecifics for optimal flower-visiting behavior [30].

The effects of predators, conspecific or heterospecific pollinators on the flower-visiting behavior of pollinators have been extensively explored in recent years. However, the majority of studies have been done on Hymenoptera [18]. The effects of these factors on another important pollinator taxa, Lepidoptera, are relatively unknown. Compared with bees, butterfly species have different types of innate and learned preferences for floral traits, including color, shape, size, odor, and amino acids in nectar [4,31]. Also, butterfly and bee pollinators have different pollination niches [32]. Some studies have reported that butterflies avoid flowers with predator cues such as the presence of artificial spiders and models of spiders’ forelimbs [16,18,21]. However, whether and how resident butterflies affect butterflies’ visitations to flowers with and without the presence of predator cues is unknown. Here, we tested the effects of predators, conspecific or heterospecific residents on the flower-visiting behavior of butterfly species by observing the behavioral response of butterflies reared in captivity under semi-natural conditions in the butterfly pavilion of Tama Zoological Park.

Thousands of butterflies are released throughout the year in the butterfly pavilion of the insectarium at Tama Zoological Park (Tokyo, Japan). All adult butterflies fly freely in the pavilion. The pavilion provides several benefits for experimental studies of the flower-visiting behavior of butterflies. First, the pavilion has stable environmental conditions (e.g. temperature) with minimal impact from natural disturbances such as wind and rain. Second, a number of flower-visiting behaviors of many butterfly species can be easily observed in one location. Therefore, we can compare variations in visiting behavior between species and examine how wing size variation of butterfly species affect their behavioral responses to predators and resident butterflies. Third, most individuals in the butterfly pavilion were reared in captivity and had no (or little) experience with predators. Although a few predators (mantises and spiders) manage to enter the pavilion, they are evicted as soon as they are discovered. Therefore, the butterflies in the pavilion can be regarded as displaying an almost exclusively innate response to predators on flowers.

In this study, we set up a dual choice experiment in the butterfly pavilion to explore the effects that the presence of predators and resident pollinators have on the flower-visiting behavior of butterflies. Specifically, we addressed following questions: (1) Do predator cues near an artificial flower and wing size of the visiting butterfly affect the flower-visiting behavior of butterflies? (2) Do the live resident butterfly and wing size differences between the resident and the visiting butterfly affect the flower-visiting behavior of butterflies? (3) How does the spatial relationship of predator cues and resident butterflies change the flower-visiting behavior of butterflies?

## Materials and Methods

### Experimental location and settings

**Fig 1:**
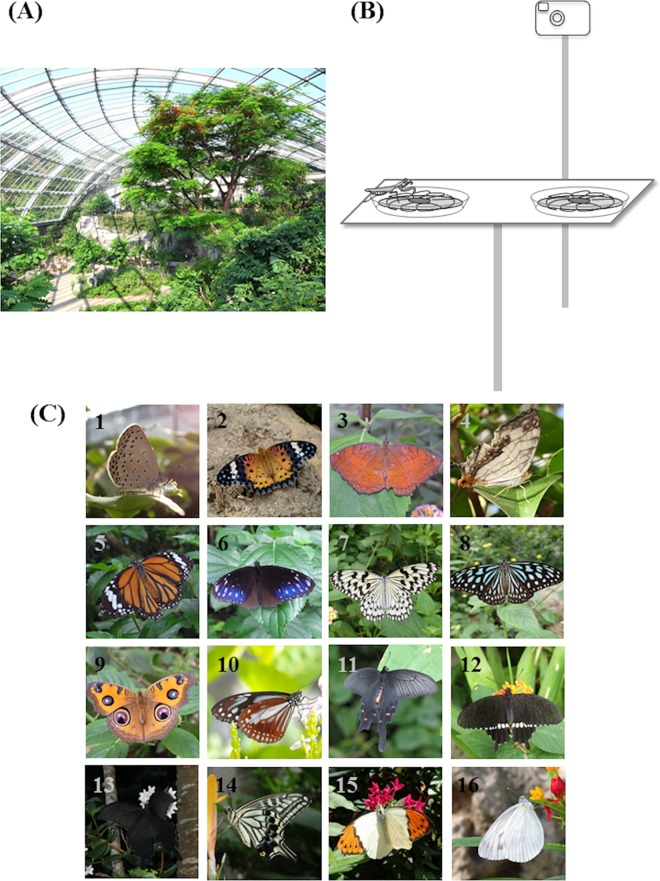
**(A) The butterfly pavilion at Tama Zoological Park, Hino city, Tokyo, Japan. (B) Schematic diagram of experimental setup. (C) Butterfly species observed in the experiment.** 1, *Pseudozizeeria maha;* 2, *Argyreus hyperbius;* 3, *Ariadne ariadne;* 4, *Cyrestis thyodamas;* 5, *Danaus genutia;* 6, *Euploea mulciber;* 7, *Idea leuconoe;* 8, *Ideopsis similis;* 9, *Junonia almana;* 10, *Parantica sita;* 11, *Byasa alcinous;* 12, *Papilio polytes;* 13, *Papilio protenor;* 14, *Papilio xuthus;* 15, *Hebomoia glaucippe;* 16, *Pieris melete*.

All experiments were performed in the butterfly pavilion at Tama Zoological Park, Hino city, Tokyo, Japan by permission of Tama Zoological Park. The pavilion has a total floor space of 1140 m^2^ and a maximum ceiling height of 16 m and is covered by a glass roof (Fig 1A). In the 5-month experimental period (Mar–July, 2015) and the 3-month pre-experimental period (Dec–Feb, 2014/15), adult butterflies of 23 species were released into the pavilion. The number of butterflies released each month, larval feeding plants, and the estimated number of generation in captive breeding for each species are included in S1 Table. Many plants, including *Ficus microcarpa*, *Ficus virgata*, *Cassia surattensis*, *Lantana camara*, *Pentas lanceolata*, and *Stachytarpheta dichotoma*, are grown in the pavilion as nectar sources and/or roosts for butterflies. The temperature is controlled by a hot water heating system and by opening or closing windows. All adult butterflies are kept under a natural day/night regime.

Two experimental sites were established 2 m away from the pavilion walkways to minimize the disturbance by visitors. At each site, we set two artificial flowers at opposite ends of a wooden board (38 cm × 11 cm × 1 cm). The height of the board from the ground was approximately 100 cm. Artificial flowers were included in the study rather than real flowers to be able to control the flower characters (e.g. shape, color, nectar and odor). The artificial flowers were created by arranging a flower-shaped yellow nylon on sponge (9 cm in diameter) in a white plastic dish (10 cm in diameter and 2 cm deep). The sponge was soaked in 10% sugar-water. The two artificial flowers were separated from one another by a center-to-center distance of 20 cm (Fig 1B). The artificial flowers and sponges were washed and soaked every day.

### Experimental procedures

To examine the effects of predators and resident butterflies on the flower-visiting behaviors of butterflies, we performed two types of choice experiments: a predator experiment and a no-predator experiment. In the predator experiment, a dead mantis, *Tenodera aridifolia*, was set to the side of one of the two artificial flowers. The mantis was collected in the pavilion and dried at room temperature. We changed the position of the mantis every day to randomize the effect of the position. The mantis was positioned so that the head was located over the center of the artificial flower and the rear of the abdomen was facing away from the other artificial flower (Fig 1B). In the no-predator experiment, we did not put any object on the artificial flowers. The predator and the no-predator experiments were performed for 14 days (from March 6 to May 13) and 17 days (from May 15 to July 7), respectively. Video recording was done only during sunny conditions.

We recorded the flower-visiting behavior of butterflies between 0800 and 1300 with a digital video camera (Ricoh, WG-20). One recording period was approximately 84 minutes due to the camera’s memory capacity, and recording was carried out twice a day at each experimental site. Video was recorded at 15 frames per second at a spatial resolution of 320 × 240 pixels. The camera was located approximately 120 cm away at an upward angle from the artificial flowers. After excluding data that contained any artificial disturbance by the pavilion’s keepers, 4853 minutes for the predator experiment and 5530 minutes for the no-predator experiment were used for the analysis. It was not possible to score the data blind because our study involved focal animals in the field.

### Data classification

**Fig 2:**
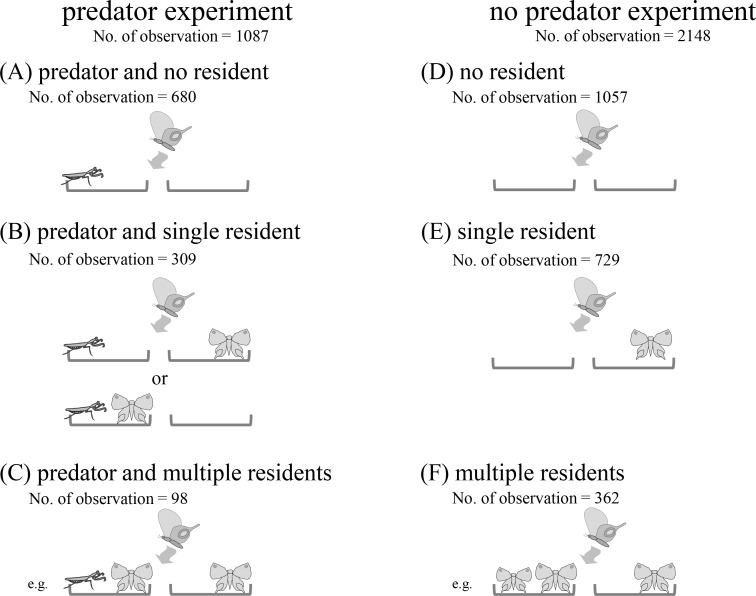
Schematic figures of data classification in the predator (A-C-) and the no-predator experiments (D-F).

For the statistical analyses, we identified the species of the visiting butterflies and classified all visiting behaviors observed in the predator experiments into three categories depending on the number of resident butterflies (Fig 2): “predator and no resident”, i.e., visiting in a situation where the predator is located on one side with no resident butterfly; “predator and single resident”, i.e., visiting in a situation where the predator is located on one side and a resident is located on either the same or opposite side to the predator; and “predator and multiple residents”, i.e., visiting in a situation where the predator is located on one side and multiple residents are located on either side or both sides. In the same way, we classified the visiting behaviors of the no-predator experiment into three categories: “no resident”, i.e., visiting in a situation without any resident butterfly; “single resident”, i.e., visiting in a situation where a resident butterfly is located on either side; “multiple residents”, i.e., visiting in a situation where multiple resident butterflies are located on either side or both sides or either side.

To examine whether the species variation in wing size affect a visiting butterfly’s behavioral response to the presence of predators or resident butterflies, we measured the forewing length of three to five individuals of each butterfly species. For the statistical analysis, we used averaged lengths of each butterfly species as a representative value (S2 Table). The majority of butterfly species did not show sexual dimorphism, so we were unable to identify the sex of individuals from the video recordings.

### Statistical analyses

To investigate the factors affecting the flower-visiting behavior of butterfly species, we adopted a generalized linear mixed model (GLMM) with the visiting choice (left or right as seen from video) and time spent on the artificial flower as response variables and species ID and setup (experimental site) as the random effect. We applied a binomial distribution for the analysis of visiting choice and a Gaussian distribution for the analysis of time spent on the flower. The models were fitted by using the lmer function in the “lme4” package in R. For all statistical analyses, we used the likelihood ratio test to evaluate the significance of the explanatory variables. We excluded the visiting data of butterflies that had not flown away from the artificial flower at the time recording was terminated.

To investigate how the presence of predators and the wing size of the visiting butterfly species affected the flower-visiting behavior, we analyzed the data of “predator and no resident” category (Fig 2A). In the analysis, we used the location of the predator, the wing size of the visiting butterfly, and their interaction as explanatory variables. To investigate how the presence and characters of a resident butterfly affected the flower-visiting behavior, we analyzed the data of “single resident” category (Fig 2E) and used the presence of the resident, type of resident butterfly (conspecific or heterospecific with visiting butterfly), wing size difference between visitor and resident species and their interactions as explanatory variables. To investigate the combined effects of a predator and a resident butterfly on flower-visiting behavior, we analyzed the data of “predator and single resident” category (Fig 2B). We used presence of predator, presence of resident, type of resident (conspecific or heterospecific), wing size of visiting species and wing size difference between visitor and resident species and their two- and three-way interactions as explanatory variables. If no significant effect of interactions were detected, the interaction terms were removed from the model to create the final model. Then we evaluated the significance of the explanatory variables in the final model. The data of “predator and multiple residents” category (Fig 2C), “no resident” category (Fig 2D) and “multiple residents” category (Fig 2F) were not used for analysis.

## Results

In total, 3235 visitations by 16 butterfly species (1087 for the predator experiment and 2148 for the no-predator experiments) were observed (Fig 1C, S2 Table). For the predator experiment, we classified 680 visitations as “predator and no resident”, 309 visitations as “predator and single resident”, and 98 visitations as “predator and multiple resident”. For the no-predator experiment, we classified 1057 visitations as “no resident”, 729 visitations as “single resident”, and 362 visitations as multiple residents (Fig 2).

### The effects of dead predator and the wing size

**Fig 3:**
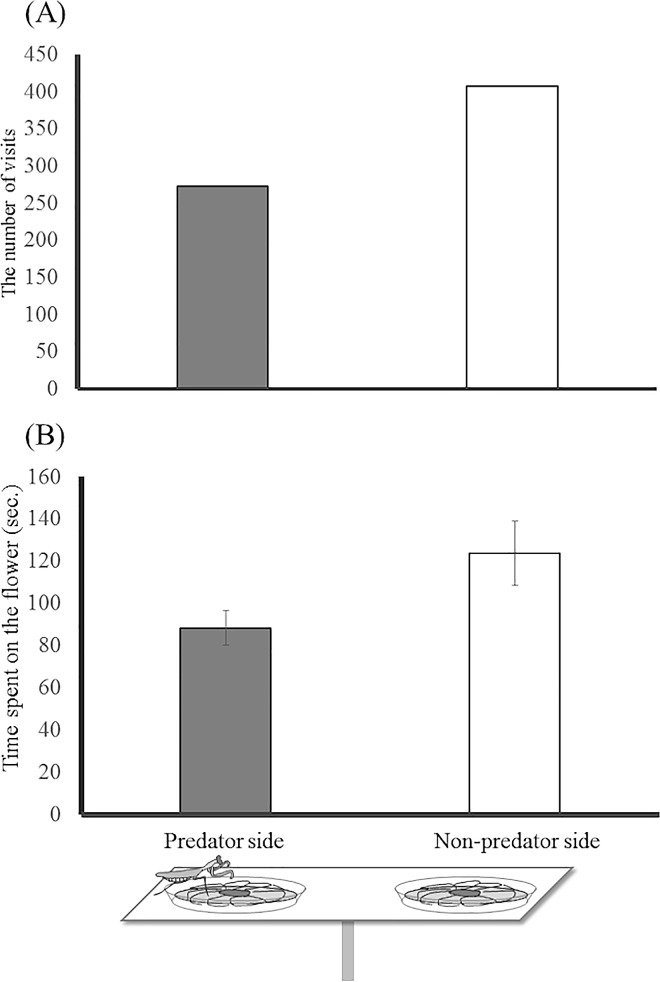
(A) The number of visits and (B) time spent on the artificial flower on the side occupied or not occupied by a dried specimen of *Tenodera aridifolia* (mean ± SE).

The presence of the dead mantis affected the visiting behavior of butterflies (Table 1). Butterflies significantly avoided visiting the predator side (Fig 3A) and spent moderately less time on that side (Fig 3B). Wing size of the visiting butterfly did not affect the visitor’s response to the predator (Table 1).

**Table 1:** Effects of presence of dead predator, wing size of the visiting butterfly species and the interaction on the visit choice and the time spent on the flower.

Response variables	Explanatory variables	d.f	LR-stat (Deviance)	P-value
Visit choice				
	Final model			
	Predator presence	1	25.942	**<0.01**
	Wing size of visitor	1	0.0145	0.90
	Removed interaction term			
	Predator presence × Wing size	1	0.2499	0.62
Time spent on the flower				
	Final model			
	Predator presence	1	3.0468	0.08
	Wing size of visitor	1	0.5216	0.47
	Removed interaction term			
	Predator presence × Wing size	1	1.8679	0.17

### The effects of resident butterfly and the wing size

**Fig 4:**
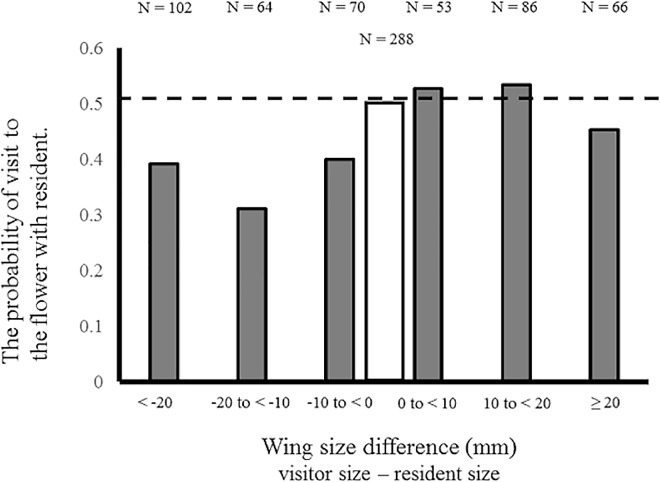
The probability of visit to an artificial flower occupied by a resident. Filled bar represents the average probability in each class of wing-size differences between the visitor and the resident butterfly species. Open bar represents the probability of visit to the flower occupied by a conspecific resident (wing size difference is zero).

The type of resident species (i.e., conspecific or heterospecific with visitor) and interaction between the location of and type of resident butterfly did not affect the visiting choice and time spent on the flower (Table 2). On the other hand, the difference in wing size between the visitor and the resident species had a significant effect on the visiting avoidance (interaction between resident presence × wing size difference as shown in Table 2). The visiting butterfly avoided visiting the artificial flower occupied by the resident species when the wing size of the resident was larger than that of the visitor (Fig 4). The presence of resident butterfly, type of resident species and wing size difference between the visitor and the resident species did not affect the time spent on the flower.

**Table 2:** Effects of presence of resident butterfly, type of resident butterfly (conspecific or heterospecifics with visitor), wing size difference between visitor and resident butterfly species and the interactions on the visit choice and the time spent on the flower.

Response variables	Explanatory variables	d.f	LR-stat (Deviance)	P-value
visit choice				
	Final model			
	Resident presence	1	5.924	
	Wing size difference	1	0.133	
	Type of resident	1	0.409	0.522
	Resident presence × Wing size difference	1	5.255	**0.022**
	Removed interaction terms			
	Resident presence × Types of resident	1	2.277	0.131
Time spent on the flower				
	Final model			
	Resident presence	1	1.889	0.169
	Type of resident	1	0.088	0.767
	Wing size difference	1	0.101	0.751
	Removed interaction terms			
	Resident presence × Types of resident	1	0.397	0.529
	Resident presence × Wing size difference	1	1.011	0.315

### The effects of coexistence of dead predator and resident butterfly

**Fig 5:**
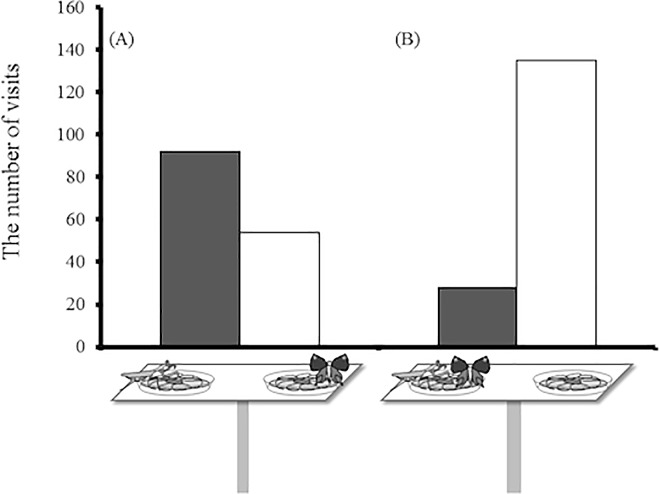
The number of visits to each artificial flower (A) when the resident butterfly is located on the side opposite the dried specimen of *Tenodera aridifolia* and (B) when the resident butterfly is located on the same side as the dried specimen of *T*. *aridifolia*.

The interaction between the presence of the predator and the presence of the resident had a significant effect on the visiting choice (the interaction between predator presence and resident location in Table 3). When the resident was located on the side opposite the predator, butterflies avoided visiting the resident side rather than avoid visiting the predator side (Fig 5A). In contrast, when the resident was located on the same side as the predator, the visiting butterfly strongly avoided visiting the side occupied by both the resident and predator and preferred to visit the empty side (Fig 5B). The type of resident butterfly, wing size of visiting species, wing size difference between the visitor and the resident species and the other interaction terms had no effect on the visiting choice (Table 3). In this analysis, none of the variables affected the time spent on the flower (Table 3).

**Table 3:** Effects of presence of dead predator, presence of resident butterfly, type of resident butterfly (conspecific or heterospecifics), wing size of the visiting butterfly species, wing size difference between visitor and resident butterfly species and their interactions on the visit choice and the time spent on the flower.

Response variables	Explanatory variables	d.f	LR-stat (Deviance)	P-value
Visit choice				
	Final model			
	Predator presence	1	7.935	
	Resident presence	1	14.742	
	Type of resident butterfly	1	0.16	0.689
	Wing size of visitor	1	0.499	0.480
	Wing size difference	1	0.269	0.604
	Predator presence × Resident presence	1	61.301	**< 0.01**
	Removed interaction terms			
	Predator presence × Type of resident butterfly	1	0.534	0.465
	Resident presence × Type of resident butterfly	1	0.657	0.418
	Predator presence × Resident presence × Type of resident butterfly	1	2.549	0.110
	Predator presence × Wing size of visitor	1	0.019	0.890
	Resident presence × Wing size difference	1	3.33	0.068
Time spent on the flower				
	Final model			
	Predator presence	1	0.15109	0.698
	Resident presence	1	0.024	0.877
	Type of resident butterfly	1	1.27467	0.259
	Wing size of visitor	1	0.15921	0.690
	Wing size difference	1	0.19173	0.662
	Removed interaction terms			
	Predator presence × Resident presence	1	0.00654	0.936
	Predator presence × Type of resident butterfly	1	0.01202	0.913
	Resident presence × Type of resident butterfly	1	1.13322	0.287
	Predator presence × Resident presence × Type of resident butterfly	1	0.09181	0.762
	Predator presence × Wing size of visitor	1	1.1605	0.281
	Resident presence × Wing size difference	1	0.0333	0.855

## Discussion

In this study, we examined the effects of the presence of a dead predator and resident butterflies on the flower-visiting behavior of 16 butterfly species using dual choice experiment under semi-natural conditions in a butterfly pavilion. Firstly, we found that visiting butterflies avoided the flower with the dead predator. Secondly, visiting butterflies avoided the resident butterflies larger than the visitor. These results are consistent with the behaviors of bee pollinators [11,18,28,33,34]. Finally, butterflies showed greater avoidance of the predator when a resident was present together with the predator.

We observed that visiting butterflies avoided the flower with the dead predator (mantis). However, our experimental setting could not determine whether the butterflies truly perceived the dead mantis as a predatory cue or whether they were merely avoiding any foreign object on the flower. Previous studies have suggested that Hymenoptera pollinators portray avoidance response to any object on flowers rather than avoidance of the predator per se [18]. In addition, Gonçalves-Souza (2008) demonstrated that flowers of *Rubus rosifolius* containing a spider replica or a sphere simulating a spider abdomen were visited less frequently by Lepidoptera pollinators than control flowers were under natural conditions. Considering the morphological variation of predators and high risk of missing the predator, avoidance response to any foreign object on flowers might be adaptive for insect pollinators. Therefore, the avoidance response to the dead mantis by the butterflies might be induced by the avoidance to any foreign object on flowers. Further experiments are required to determine what types of visual signals induce butterfly avoidance. The majority of butterflies in the pavilion did not have any experience of predation because there are few flower-dwelling predators in this environment. Thus, avoidance behavior might be attributed to an innate response rather than experience with predator attacks. The degree of avoidance shown by the butterflies in this study seemed to be weaker than that shown by wild butterflies in previous studies [16,18,21]. For example, a meta-analysis of the wild pollinator population revealed that signals of predation significantly decreased flower visitation rates by 36% and time spent on flowers by 51% [18]. Because the risk of being attacked by predators would be greater for wild butterflies than for the butterflies in the pavilion, the strong avoidance response reported in the wild might reflect a learned response to prior predation experiences.

Gonçalves-Souza et al. (2008) suggested that unpalatable or toxic butterflies might have no or little avoidance response to the predator cues. In this study, we could not test the effect of toxicity on the avoidance behavior because of the high collinearity between the wing size and the potential toxicity (large butterflies tended to be toxic, S2 Table). However, the wing size of visiting butterflies did not affect the visiting behavior in response to the dead predator. This result suggests that the toxicity of the visiting butterfly also had little effect on the visiting choice and time spent on flower of the butterflies.

We found that the size difference between visiting and resident butterflies affected the flower-visiting behavior of butterflies: smaller butterflies avoided visiting the flower occupied by larger residents. Size-based dominance relationships among flower-visiting insects have been reported for bee and hoverfly species [28,33,34]. Our results indicate that a size-based dominance relationship also affects the visiting behavior of butterfly pollinators. Contact with other butterflies, especially for small butterflies, might damage their wings, and result in reduction of flight ability and sexual attractiveness. In the butterfly pavilion, the butterflies live in high densities and nectar sources are sometimes crowded with several butterflies. In this situation, the butterflies might learn the risks associated with approaching resident butterflies on flowers that are larger than they are on flowers. Future experiments will be needed to examine the size based dominance among wild butterfly pollinators.

Many species of adult butterflies have been observed to engage in mud-puddling behavior, aggregating at moist ground to feed on essential nutrients such as sodium. Otis et al. (2006) reported that two *Papilio* butterflies were highly attracted to artificial puddles with dead decoys of conspecific and heterospecific butterflies [35]. In contrast, our results showed that flower-visiting butterflies were not attracted to conspecific or heterospecific residents. Compared with the nutrients in the ground, flower nectar may be a limited resource for butterfly species.

When the resident butterfly was located on the opposite side to the predator, the visiting butterfly avoided the resident side and preferred the predator side (Fig 5A). This response is somewhat strange because the predator must be more “dangerous” than the resident butterfly. One possible explanation is that the resident butterfly might be more conspicuous than the dead predator to the visiting butterfly because resident butterflies often opened and closed their wings on the artificial flower. Another possibility is that while butterflies in the pavilion have learned the risk of approaching resident butterflies on flowers, the butterflies have not learned the risk of approaching predators. In either case, this result implies that the visiting butterflies did not perceive the dead mantis as a dangerous predator, but rather as a foreign object on the flower. On the other hand, butterflies showed greater avoidance of a predator when the predator was present together with the resident butterfly than when the predator was located on the side opposite the resident butterfly (Fig 5B). This result suggests that the combination of the dead predator and the resident living butterfly can be strong signals to avoid visiting the flower. Although the mechanism behind the strong avoidance of the combined cues of predator and resident butterfly is unclear, the presence of resident butterflies might emphasize the signal of the predator (or vice versa) for flower-visiting butterflies

Our dual choice experiments focused on the local-scale response of flower-visiting butterflies to predators and resident butterflies. However, the broad-scale response of pollinators is also important for the understanding of the effect of predators and residents on the behavior of pollinators in the context of community ecology. Although the present study could not directly compare the data between the predator and no-predator experiments because the periods of the two experiments differed, the visitation rate (total number of visitations / total recorded time) and the species compositions of visiting butterflies seemed to differ between the no-predator and predator experiments (S2 Table). These differences might be accounted for by the broad-scale response of butterflies to the existence of the predator. Future studies are required to determine the effect of predators on the broad-scale response of insect pollinators. It is difficult to observe a number of butterfly pollination events under field conditions. The present study indicates that experimental tests in a butterfly pavilion might be a useful approach for studies of butterfly behavior.

## Supporting Information

S1 Table Species list, description, and the number of released adults of each butterfly species in the butterfly pavilion at Tama Zoological Park. (XLSX)

S2 Table Abundance, composition, wing size and toxicity of butterflies observed in the predator and the non-predator experiments. (XLSX)
